# Prevalence and zoonotic potential of *Enterocytozoon bieneusi* and *Giardia duodenalis* in dairy cattle from Anhui Province, China

**DOI:** 10.3389/fvets.2025.1613342

**Published:** 2025-06-05

**Authors:** Falei Li, Shimei Cheng, Chaoyue He, Libing Meng, Aihong Wang, Meng Shao, Gaoxiao Xu, Huilin Zhang

**Affiliations:** Anhui Province Key Laboratory of Embryo Development and Reproductive Regulation, College of Biological and Food Engineering, Fuyang Normal University, Fuyang, China

**Keywords:** *Enterocytozoon bieneusi*, *Giardia duodenalis*, dairy cattle, zoonosis, China

## Abstract

**Introduction:**

*Enterocytozoon bieneusi (E. bieneusi)* and *Giardia duodenalis (G. duodenalis)* are common intestinal pathogens in humans and farmed animals. There is limited data available on the positivity rates and genetic identity of *E. bieneusi* and *G. duodenalis* in dairy cattle from Anhui, China.

**Methods:**

To understand the transmission of *E. bieneusi* and *G. duodenalis* in these animals, a total of 1,043 fecal samples were collected from cattle on five farms (Fuyang, Huainan, Huaibei, Bengbu, and Jieshou) in Anhui province of China between May 2023 and August 2024. The *G. duodenalis* in fecal samples was detected by nested PCR targeting a 511-bp fragment of the *β*-giardin (*bg*) gene, a 599-bp fragment of the glutamate dehydrogenase (*gdh*) gene, and a 530-bp fragment of the triosephosphate isomerase (*tpi*) gene. The *E. bieneusi* was detected by nested PCR targeting a 392-bp fragment of the internal transcribed spacer (ITS) of the rRNA gene.

**Results:**

The PCR analysis revealed positivity rates of 29.7% (310/1043) for *E. bieneusi* and 2.8% (29/1043) for *G. duodenalis*. The cattle from the Bengbu farm had significantly higher positivity rates of *E. bieneusi* than Fuyang, Huainan, and Huaibei farms (*χ*^2^ = 61.6, *df* = 1, *p* < 0.0001; *χ*^2^ = 76.4, *df* = 1, *p* < 0.0001; *χ*^2^ = 20.6, *df* = 1, *p* < 0.0001). A total of 11 known genotypes of *E. bieneusi* have been identified: J (*n* = 154), BEB4 (*n* = 76), I (*n* = 63), CGC1 (*n* = 8), N (*n* = 2), BEB8 (*n* = 2), ALP1 (*n* = 1), BLC13 (*n* = 1), CHC13 (*n* = 1), CHN6 (*n* = 1), and D (*n* = 1). Additionally, two genotypes of *G. duodenalis* have been identified, including assemblage A (*n* = 6) and assemblage E (*n* = 23).

**Discussion:**

The results indicate that known zoonotic *E. bieneusi* and *G. duodenalis* are prevalent in dairy cattle, thereby enhancing our understanding of the genetic diversity and transmission of these pathogens in these animals.

## 1 Introduction

*Enterocytozoon bieneusi* (*E. bieneusi*) and *Giardia duodenalis* (*G. duodenalis*) are common intestinal pathogens in humans and other farmed animals, especially cattle (1, 2). The life cycle of *E. bieneusi* and *G. duodenalis* consists of two main stages. Spores of the former and cysts of the latter are ingested by susceptible hosts, invade the epithelial cells that line the intestinal lumen, and replicate intracellularly (3, 4). Diarrhea, malnutrition, and weight loss are the primary symptoms of giardiasis and microsporidiosis (5, 6). These two zoonotic pathogens cause outbreaks of diarrhea diseases in both humans and animals worldwide (7, 8).

At least 500 genotypes of *E. bieneusi* and 11 major genetic groups have been identified by conducting phylogenetic analysis of nucleotide sequences of the internal transcribed spacer (ITS), with the genotypes of Group 1 being the major zoonotic pathogens (1, 4, 5). A large number of studies indicate that cattle serve as a prevalent reservoir host for *E. bieneusi* (4, 5). Genotypes BEB4, BEB6, I, and J in Group 2 are frequently reported in cattle (5, 8–12). The genotypes BEB6 and BEB4 are more common in cattle. Genotypes I and J have been found in a wide range of ruminants (5). While they are predominantly reported in ruminants, genotypes BEB4, BEB6, I, and J have been shown to have the capacity to infect humans (13–15). Therefore, *E. bieneusi* genotypes in cattle possess zoonotic potential.

To date, a total of eight assemblages (A-H) of *G. duodenalis* have been identified through sequence analysis of *β*-giardin (*bg*), triosephosphate isomerase (*tpi*), and glutamate dehydrogenase (*gdh*) genes (7, 16). Of the eight assemblages of *G. duodenalis,* assemblages A and B have the widest host ranges, including humans and cattle (17). However, assemblages C-H of *G. duodenalis* are host-specific (18). The most common genotype of *G. duodenalis* in cattle is the assemblage E, whereas assemblages A and B occur sporadically (7). Assemblage A of *G. duodenalis* is responsible for the majority of cases of giardiasis in humans (7). Recent studies indicate that more than 50 cases of human giardiasis attributed to assemblage E have been reported in Egypt, New Zealand, Brazil, Vietnam, and Australia (6, 19–22). Therefore, there is a zoonotic potential for assemblages of *G. duodenalis* in bovine animals.

Bovine animals are widely farmed in Anhui, China, which is a large agricultural province. To date, only a limited number of studies have been carried out to determine the identity of *G. duodenalis* and *E. bieneusi* in cattle. To understand the transmission of *E. bieneusi* and *G. duodenalis* in these animals, we examined the prevalence and genetic identity of *G. duodenalis* and *E. bieneusi* in dairy cattle in Anhui, China. The data show a prevalent occurrence of zoonotic genotypes in these animals. The aim of the present study was to investigate the distribution and genetic identity of two pathogens in cattle in Anhui, China. The data obtained suggest that the zoonotic genotypes of both pathogens are commonly found in these animals.

## 2 Materials and methods

### 2.1 Specimens

A total of 1,043 fecal samples were collected from cattle on five farms (Fuyang, Huainan, Huaibei, Bengbu, and Jieshou) in Anhui province of China between May 2023 and August 2024. Farms in Bengbu, Huaibei, Huainan, and Fuyang were established in 2011, 2018, 2021, and 2023, respectively; however, the year of establishment for Jieshou Farm was unclear. Depending on the number of animals on the farm, 234, 167, 238, 309, and 95 were randomly collected from Fuyang, Huainan, Huaibei, Bengbu, and Jieshou, respectively. These cattle did not originate from the tested areas and were imported from the Netherlands. All fecal samples were obtained from intensive dairy farming systems, and all cattle exhibited no obvious clinical signs during the study period. All cattle were housed separately according to their age, with calves under 2 months housed separately. The cattle were divided into four age groups, based on their age: < 2 months (*n* = 323), 2–6 months (*n* = 237), 6–12 months (*n* = 249), and >12 months (*n* = 234). All fecal samples were collected from the rectum of each animal, and the age of cattle, the time, and place of the sample collection was recorded. All samples were stored in 2.5% potassium dichromate at 4°C prior to use in the DNA extraction.

### 2.2 DNA extraction

Approximately 200 mg fecal samples from cattle were washed three times with distilled water by centrifugation. Genomic DNA was extracted using the E. Z. N. A.®Stool DNA Kit (Omega Biotek Inc., Norcross, GA, USA). The extracted genomic DNA was stored at −20°C before being used in PCR analysis.

### 2.3 Detection of *G. duodenalis* and *E. bieneusi*

Assemblages of *G. duodenalis* in fecal samples were detected by nested PCR targeting a 511-bp fragment of the *β*-giardin (*bg*) gene, a 599-bp fragment of the glutamate dehydrogenase (*gdh*) gene, and a 530-bp fragment of the triosephosphate isomerase (*tpi*) gene (23–25). In contrast to *G. duodenalis*, *E. bieneusi* was detected by nested PCR targeting a 392-bp fragment of the ITS of the rRNA gene (26). Each PCR assay included both positive and negative samples. Two replicates were used for PCR analysis of each sample at each locus.

### 2.4 Sequence analysis

All positive products of the second PCR were sequenced bi-directionally on an ABI 3730 Auto sequencer (Applied Biosystems, Foster City, CA, USA) to identify the presence of *G. duodenalis* and *E. bieneusi*. The nucleotide sequences were assembled using ChromasPro 2.1.5.0,^1^ edited using BioEdit 7.1.3.0,^2^ and aligned using ClustalX 2.0.11.^3^ The phylogenetic relationship of the *E. bieneusi* was analyzed using maximum likelihood analysis implemented in Mega 7.0.^4^

### 2.5 Statistical analysis

The chi-squared test, implemented in SPSS 20.0 (IBM Inc., Chicago, IL, USA), was performed to compare positivity rates of *G. duodenalis* and *E. bieneusi* between geographical areas and age groups. Comparisons of positivity rates were made using the Mann–Whitney U test, and the *p*-values were calculated. Differences with *p* ≤ 0.05 were considered significant.

## 3 Results

### 3.1 Distribution of genotypes of *E. bieneusi* and *G. duodenalis* in cattle

The PCR analysis revealed that the positivity rates of *E. bieneusi* from five farms was 29.7% (310/1043; Table 1). The positivity rates of *E. bieneusi* were 15.8% (37/234), 8.4% (14/167), 29.4% (70/238), 47.9% (148/309), and 43.2% (41/95) in Fuyang, Huainan, Huaibei, Bengbu, and Jieshou, respectively. Thus, cattle from Bengbu had significantly higher positivity rates of *E. bieneusi* than Fuyang, Huainan, and Huaibei cities (*χ*^2^ = 61.6, *df* = 1, *p* < 0.0001; *χ*^2^ = 76.4, *df* = 1, *p* < 0.0001; *χ*^2^ = 20.6, *df* = 1, *p* < 0.0001). Conversely, no significant difference was found in the positivity rates of *E. bieneusi* between Bengbu and Jieshou (*χ^2^* = 0.66, *df* = 1, *p* = 0.4182; Table 1). By age, the positivity rates of *E. bieneusi* were 14.8% (48/323), 49.8% (118/237), 30.5% (76/249), and 29.1% (68/234) in < 2 months animals, 2–6 months animals, 6–12 months animals, and > 12 months animals, respectively. Furthermore, 2–6 months cattle had significantly higher positivity rate of *E. bieneusi* than < 2 months animals, 6–12 months animals, and > 12 months animals (*χ*^2^ = 80.2, *df* = 1, *p* < 0.0001; *χ*^2^ = 18.8, *df* = 1, *p* < 0.0001; *χ*^2^ = 21.2, *df* = 1, *p* < 0.0001; Table 2).

**Table 1:** Distribution of *Enterocytozoon bieneusi* genotypes in cattle on farms from Anhui province, China.

Location	Age (months)	No. specimens	No. positive (%)	*p*-value	*Enterocytozoon bieneusi*										
					ALP1	BEB4	BEB8	BLC13	CGC1	CHC13	CHN6	D	I	J	N
Fuyang	< 2	215	30 (14.0)	*p* < 0.0001	–	4	–	–	1	–	–	–	8	17	–
	2–6	19	7 (36.8)		–	3	–	–	–	–	–	–	2	2	–
	subtotal	234	37 (15.8)		–	7	–	–	1	–	–	–	10	19	–
Huainan	< 2	35	1 (2.9)	*p* < 0.0001	–	–	–	–	–	–	–	–	–	1	–
	2–6	43	6 (14.0)		–	–	–	–	–	–	–	–	–	6	–
	6–12	69	6 (8.7)		–	1	–	–	–	–	–	–	–	3	2
	> 12	20	1 (5.0)		1	–	–	–	–	–	–	–	–	–	–
	subtotal	167	14 (8.4)		1	1	–	–	–	–	–	–	–	10	2
Huaibei	< 2	19	12 (63.2)	*p* < 0.0001	–	1	–	1	–	–	–	1	1	8	–
	2–6	31	6 (19.4)		–	4	–	–	2	–	–	–	–	–	–
	6–12	122	48 (39.3)		–	17	–	–	2	–	1	–	4	24	–
	>12	66	4 (6.1)		–	1	–	–	–	–	–	–	–	3	–
	subtotal	238	70 (29.4)		–	23	–	1	4	–	1	1	5	35	–
Bengbu	< 2	54	5 (9.3)	–	–	–	–	–	–	–	–	–	1	4	–
	2–6	144	99 (68.8)		–	29	–	–	–	–	–	–	9	61	–
	6–12	58	22 (37.9)		–	7	–	–	–	–	–	–	6	9	–
	> 12	53	22 (41.5)		–	3	2	–	1	–	–	–	11	5	–
	subtotal	309	148 (47.9)		–	39	2	–	1	–	–	–	27	79	–
Jieshou	> 12	95	41 (43.2)	*p* = 0.4182	–	6	–	–	2	1	–	–	21	11	–
Total		1,043	310 (29.7)	–	1	76	2	1	8	1	1	1	63	154	2

**Table 2:** Occurrence of *Enterocytozoon bieneusi* genotypes in cattle on farms from Anhui province, China, broken down by age.

Age (months)	No. specimens	No. positive (%)	*p-*value	*Enterocytozoon bieneusi*										
				ALP1	BEB4	BEB8	BLC13	CGC1	CHC13	CHN6	D	I	J	N
< 2	323	48 (14.8)	*p* < 0.0001	–	5	–	1	1	–	–	1	10	30	–
2–6	237	118 (49.8)	–	–	36	–	–	2	–	–	–	11	69	–
6–12	249	76 (30.5)	*p* < 0.0001	–	25	–	–	2	–	1	–	10	36	2
> 12	234	68 (29.1)	*p* < 0.0001	1	10	2	–	3	1	–	–	32	19	–
Total	1,043	310 (29.7)	–	1	76	2	1	8	1	1	1	63	154	2

The PCR analysis revealed a positivity rate of *G. duodenalis* from five farms was 2.8% (29/1043; Table 3). The positivity rates of *G. duodenalis* were 6.5% (14/234), 2.4% (4/167), 1.3% (3/238), 2.3% (7/309), and 1.1% (1/95) in Fuyang, Huainan, Huaibei, Bengbu, and Jieshou, respectively. Thus, cattle from Fuyang had significantly higher positivity rates for *G. duodenalis* than Huaibei and Bengbu cities (χ2 = 7.6, *df* = 1, *p* = 0.0059; χ2 = 4.9, *df* = 1, *p* = 0.0261). In contrast, no significant difference was found in the positivity rates of *G. duodenalis* in Huainan and Jieshou (Table 3).

**Table 3:** Distribution of *Giardia duodenalis* genotypes in cattle on farms from Anhui province, China.

Location	Age (months)	No. specimens	No. positive (%)	*p*-value	*Giardia duodenalis*	
					A	E
Fuyang	< 2	215	14 (6.5)	–	2	12
	2–6	19	0 (0.0)		–	–
	subtotal	234	14 (6.5)		2	12
Huainan	< 2	35	1 (2.9)	*p* = 0.0872	–	1
	2–6	43	3 (7.0)		–	3
	6–12	69	0 (0.0)		–	–
	> 12	20	0 (0.0)		–	–
	subtotal	167	4 (2.4)	*p* = 0.0059	–	4
Huaibei	< 2	19	3 (15.8)		–	3
	2–6	153	0 (0.0)		–	–
	6–12	28	0 (0.0)		–	–
	>12	38	0 (0.0)		–	–
	subtotal	238	3 (1.3)		–	3
Bengbu	< 2	54	2 (3.7)	*p* = 0.0261	1	1
	2–6	144	3 (2.1)		2	1
	6–12	58	1 (1.7)		–	1
	> 12	53	1 (1.9)		1	–
	subtotal	309	7 (2.3)		4	3
Jieshou	> 12	95	1 (1.1)	*p* = 0.0520	–	1
Total		1,043	29 (2.8)	–	6	23

### 3.2 Distribution of *E. bieneusi* and *G. duodenalis* genotypes in cattle

The ITS secondary PCR products from all 310 *E. bieneusi*-positive samples were successfully sequenced. A total of 11 known genotypes were identified, including J (*n* = 154), BEB4 (*n* = 76), I (*n* = 63), CGC1 (*n* = 8), N (*n* = 2), BEB8 (*n* = 2), ALP1 (n = 1), BLC13 (*n* = 1), CHC13 (*n* = 1), CHN6 (*n* = 1), and D (*n* = 1). Among these genotypes, J (154/310) was dominant over *E. bieneusi* in animals. Furthermore, genotype N, BEB8, ALP1, BLC13, CHC13, CHN6, and D were only detected in a small number of animals. The ITS sequences from 154 J samples, 76 BEB4 samples, 63 I samples, 8 CGC1 samples, 2 N samples, 2 BEB8 samples, 1 ALP1 sample, 1 BLC13 sample, 1 CHC13 sample, 1 CHN6 sample, and 1 D sample were identical to the GenBank reference sequence KU55767, MH732750, MT231513, OK416087, MN178159, OK416086, KC860908, MN758760, OR491688, MN136733, and OK117963, respectively (Table 1).

The secondary PCR products from 29 *G. duodenalis*-positive samples have been successfully sequenced. A total of two known genotypes have been identified, including assemblage A (*n* = 6) and assemblage E (*n* = 23). Among them, assemblage E was detected in cattle in all five cities; however, assemblage A was only detected in cattle from Fuyang and Bengbu cities. In this study, assemblage E was the dominant genotype of *G. duodenalis* in animals. The obtained sequences from 23 assemblage E samples and 6 assemblage A samples were identical to the GenBank reference sequences GQ337965 and MN996372, respectively (Table 3).

### 3.3 Phylogenetic relationship of *E. bieneusi* genotypes

**Figure 1:**
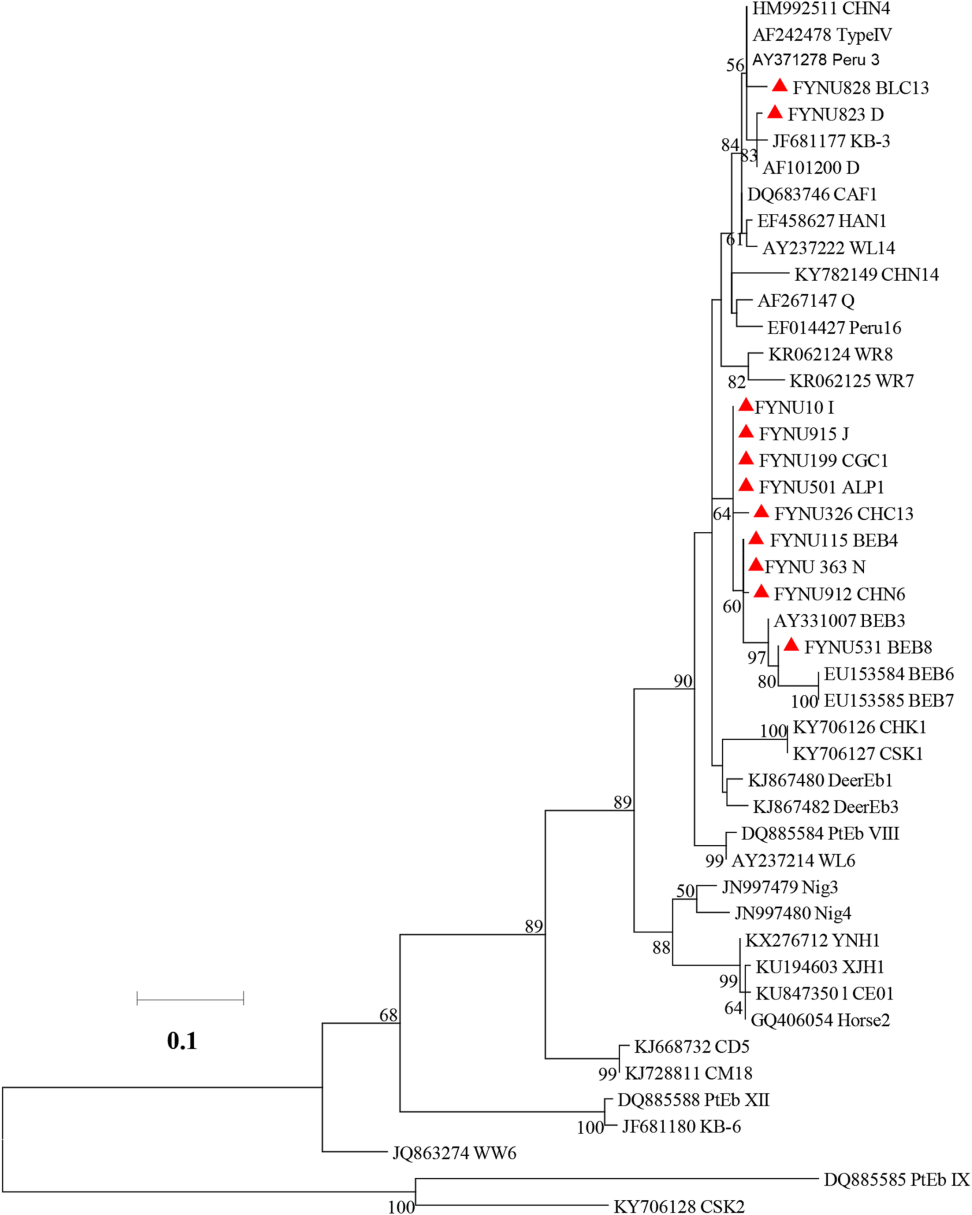
Phylogenetic relationships of *Enterocytozoon bieneusi* genotypes based on maximum likelihood analysis. The genotypes of *E. bieneusi* that have been identified in this study are indicated by red triangles. Bootstrap values below 50% are not shown. Bar = 0.02 substitutions per site.

In the maximum likelihood analysis of the ITS sequences obtained, genotypes D and BLC13 were clustered with Group 1. The other genotypes (I, J, CGC1, ALP1, CHC13, BEB4, N, CHN6, and BEB8) were clustered together with Group 2 (Figure 1).

## 4 Discussion

The results of the present study indicate that *E. bieneusi* and *G. duodenalis* are prevalent in cattle in Anhui. By sampling location, the *E. bieneusi* detection rate was found to be higher in Bengbu (47.9%, 148/309) compared to Fuyang (15.8%, 37/234), Huainan (8.4%, 14/167), and Huaibei (29.4%, 238). The long history of animal farming could have contributed to the high prevalence of *E. bieneusi* in animals in the present study. Farm patterns of long-term intensive farming lead to high animal densities and increased fecal and environmental contamination, which increase the spread of *E. bieneusi*. The *E. bieneusi* may be transmitted by other animals such as birds or rodents. By age, the detection rate (49.8%, 118/237) was significantly higher in cattle aged 2–6 months compared to other age groups, probably due to the group feeding practices of this age group of cattle. The positivity rate of *G. duodenalis*, when analyzed by sampling location and animal age, showed no significant difference between cities and animal age.

The overall detection rate of 29.7% for *E. bieneusi* is much higher than that reported in a previous study in Anhui (3.0%, 16/526), Xingjiang (16.5%, 85/514), Guangdong (15.7%, 61/388), Jiangxi (7.9%, 13/165), Yunan (0.6%, 3/490), Heilongjiang (6.5%, 75/1155), and Jiangsu (13.0%, 177/1366) (27–31). The higher detection rate of *E. bieneusi* in Anhui could also be attributed to the long history of animal farming and the presence of underage animals. The overall detection rate of 2.8% for *G. duodenalis* is much lower than that reported in a previous study from Xingjiang (24.0%, 180/749), Jiangxi (9.4%, 52/556), Yunnan (27.5%, 144/524), Taiwan (19.8%, 31/156), and Inner Mongolia (29.5%, 149/505) (32, 33–36). The lower detection rate of *G. duodenalis* in this study could also be attributed to the number of samples analyzed and the time of sampling.

The significance of *E. bieneusi* in cattle for public health is unclear. This study identified 11 distinct genotypes from 310 *E. bieneusi*-positive samples based on the ITS gene locus in cattle, which include BLC13 (*n* = 1) and D (*n* = 1), from group 1, and J (*n* = 154), as well as BEB4 (*n* = 76), I (*n* = 63), CGC1 (*n* = 8), N (*n* = 2), BEB8 (*n* = 2), ALP1 (*n* = 1), CHC13 (*n* = 1), and CHN6 (*n* = 1), from group 2. The dominant genotype of these was J (49.7%, 154/310). Genotype D of group 1 is the most common in both humans and cattle globally (4, 5, 37, 38). Genotype BLC13 was only detected in edible bullfrogs (*Lithobates catesbeiana*) in China (39). Genotypes J, I, N, BEB4, and BEB8 have been subsequently found in cattle and humans worldwide (5, 29, 40, 41). However, the genotype CHC13, which is less common, is also found in dairy cattle. In contrast, the ALP1 genotype has only been found in newborn alpacas in Peru (Unpublished data). These results suggest that cattle may be a potential source of human infection with this pathogen.

The genotypes of *G. duodenalis* detected in cattle in this study also appear to be primary zoonotic genotypes. In this study, two genotypes were identified in 29 *G. duodenali*-positive samples in cattle, including A (*n* = 6) and E (*n* = 23), with the dominant genotype being E (79.3%, 23/29). Similar to observations in other studies in recent publications, assemblages A and E were found to be the dominant genotypes in cattle (7, 32). Previous studies have shown that assemblage A is the dominant genotype in humans (2, 7). Although assemblage E is generally considered to be a host-specific genotype in bovine animals, and this genotype has been reported to occur in humans in many countries, including New Zealand, Egypt, Australia, Brazil, and Vietnam (6, 19–22). This study suggests that the presence of assemblage A and assemblage E of *G. duodenalis* in animals may have implications for public health.

## 5 Conclusion

The results of the study indicate a common occurrence of *E. bieneusi* and *G. duodenalis* in dairy cattle from Anhui, China. The results suggest that known zoonotic *E. bieneusi* and *G. duodenalis* are prevalent in dairy cattle, thereby enhancing our understanding of the genetic diversity and transmission of these pathogens in these animals. Attention should be paid to the monitoring of the spread of these zoonotic *E. bieneusi* and *G. duodenalis* in animals. To better understand the transmission of these two pathogens, future molecular epidemiological studies should involve sampling from a larger number of farms.

## Data Availability

The datasets presented in this study can be found in online repositories. The names of the repository/repositories and accession number(s) can be found at: https://www.ncbi.nlm.nih.gov/genbank/, PQ738189–PQ738196.

## Footnotes

^1^ http://technelysium.com.au/ChromasPro.html

^2^ http://www.mbio.ncsu.edu/BioEdit/bioedit.html

^3^ http://clustal.org

^4^ http://www.megasoftware.net
